# Motion Alters Color Appearance

**DOI:** 10.1038/srep36272

**Published:** 2016-11-08

**Authors:** Sang-Wook Hong, Min-Suk Kang

**Affiliations:** 1 Department of Psychology, Florida Atlantic University, FL, USA; 2 Center for Complex Systems and Brain Sciences, Florida Atlantic University, FL, USA; 3 Center for Neuroscience Imaging Research (CNIR), Institute for Basic Science (IBS), Suwon, Republic of Korea; 4 Department of Psychology, Sungkyunkwan University, Seoul, Republic of Korea

## Abstract

Chromatic induction compellingly demonstrates that chromatic context as well as spectral lights reflected from an object determines its color appearance. Here, we show that when one colored object moves around an identical stationary object, the perceived saturation of the stationary object decreases dramatically whereas the saturation of the moving object increases. These color appearance shifts in the opposite directions suggest that normalization induced by the object’s motion may mediate the shift in color appearance. We ruled out other plausible alternatives such as local adaptation, attention, and transient neural responses that could explain the color shift without assuming interaction between color and motion processing. These results demonstrate that the motion of an object affects both its own color appearance and the color appearance of a nearby object, suggesting a tight coupling between color and motion processing.

Theories of vision posit that primary processing of color and motion takes place within distinct neural pathways at early stages of visual processing^1^,^2^. However, growing evidence is incompatible with such distinction. For example, color specific neural responses are evident in motion-specific areas in the human visual cortex^3^,^4^,^5^, and perceived speed of motion is modulated by color^6^,^7^. Color information can be spatiotemporally integrated along the motion trajectory such that a moving object with alternating colors is perceived with an intermediated/mixed color^8^,^9^. Color also can spread over a motion-defined region^10^. These studies suggest close interactions of motion and color in visual processing. However, it remains unknown whether color appearance of an object is affected by motion of another object in the surrounding context.

Color appearance of an object is determined by its chromatic context as well as spectral light reflected from that object. For example, a light appearing yellowish when it is viewed alone can appear more greenish when it is surrounded by reddish appearing light, and can appear more reddish when it is presented within a greenish background. Such changes in color appearance due to chromatic context have been understood by modulations in color-specific neural responses due to adaptation to the average color (chromatic adaptation) and to the variation in color (chromatic contrast adaptation) within a visual scene. Specifically, sensitivity regulation in multiple stages of visual processing provides a detailed explanation for changes in color appearance due to chromatic adaptation^11^,^12^,^13^ and chromatic contrast adaptation^14^,^15^,^16^. An adaptation to chromatic contrast modulates responsiveness of the post-receptoral^17^,^18^ and cortical^19^,^20^,^21^ color-selective cells, so that the same object can appear differently when it is embedded within an extremely colorful environment than when it is embedded within a homogeneously colored or achromatic environment^16^,^21^. Nevertheless, a chromatic environment is not the only context modulating color appearance.

Here, we present a novel color induction triggered by simple motion in the surrounding context, demonstrating that *kinetic* context also influences objects’ color appearance. We refer to this change in appearance as a ‘motion-induced color shift’. When two identically colored objects are stationary, they appear exactly the same. However, when one of the objects starts moving around the other stationary one, the color appearance of the stationary object changes immediately (see the Supplementary Video S1). The magnitude of color induction is so compelling that it can be easily observed in large classroom settings with no sophisticated control of illumination, light intensity, and chromaticity. This motion-induced color shift cannot be explained by chromatic or chromatic contrast adaptation because the chromaticity of the stationary and the moving objects are identical. We also ruled out other alternatives explaining this color shift without positing interaction of color and motion information between the two objects. Potential mechanisms of the motion-induced color shift and its implications to perceptual organization and visual awareness are discussed.

## Results

### Color appearance shift in stationary objects induced by motion of other objects

**Figure 1:**
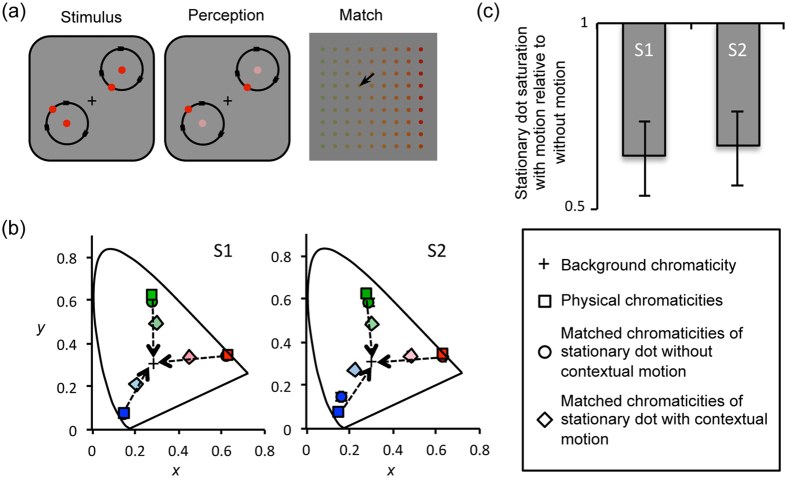
(**a**) Schematic illustration of the memory-based choice task to measure color appearance of the stationary dots. The ‘Perception’ panel illustrates the possible perceptual experience during the 2-second stimulus presentation. The ‘Match’ panel illustrates a 9 by 9 array of color patches. Observers chose the patch that matched the appearance of the stationary dot at the end of the stimulus presentation. (**b**) Results from two observers shown in CIE color space. In the diagram, the x-axis represents the x-coordinate of the CIE space, and the y-axis represents the y-coordinate of the CIE space. Squares represent the physical chromaticities of the dots (both stationary and moving). Circles, most of which are overlapped with the squares, represent the matched chromaticities of the dots without motion. Diamonds represent the matched chromaticities of the stationary dots while physically identical dots are orbiting around the stationary dots in the memory-based choice task. The cross symbol in the center represents the physical chromaticity of the background. Error bars, generally smaller than the symbol, represent ±1 standard error. (**c**) Saturation of the stationary dot with motion relative to saturation of the stationary dot without motion (in log-scale). Values lower than 1 indicate reduced saturation of the color compared to the baseline condition (color appearance without motion). Error bars represent ±1 standard error for all three colors.

In Experiment 1, we measured the shift in color appearance of a stationary object using a memory-based color choice task (see Methods). The appearance of an object with each of three primary colors (red, green and blue) was measured to reveal, first, whether the motion-induced color shift occurs for particular hues, and second, whether the direction of color shift depends on the hue. When both the stationary and moving dots had the same highly saturated red, green or blue, the appearance of the stationary dot consistently shifted toward the achromatic color (gray) irrespective of the hue; that is, it appeared de-saturated (arrows in Fig. 1b). The perceived saturation of the stationary dot with contextual motion was about 60% of that without motion in the context (Fig. 1c). The direction of color shift (de-saturation) induced by contextual motion was similar to the direction of color change due to chromatic contrast adaptation^16^, although there was no change in contextual color information.

### Can local adaptation explain the large shift in color appearance?

Is the shift in color appearance of the stationary dot indeed induced by motion-specific neural signals accompanied by a moving dot? One can posit an adaption-based account for the motion-induced color shift, which does not require interaction between stationary and moving objects. The moving dot continuously stimulates new retinotopically organized neurons, so that it is free from local adaptation. On the other hand, the stationary dot stimulates the same neural population selective to specific colors for a long period of time, which causes reduced sensitivity to the adapted colors. Thus, the motion-induced color shift may not require interaction between color and motion information, but can occur simply due to the sensitivity reduction caused by local adaptation to the stationary object.

**Figure 2:**
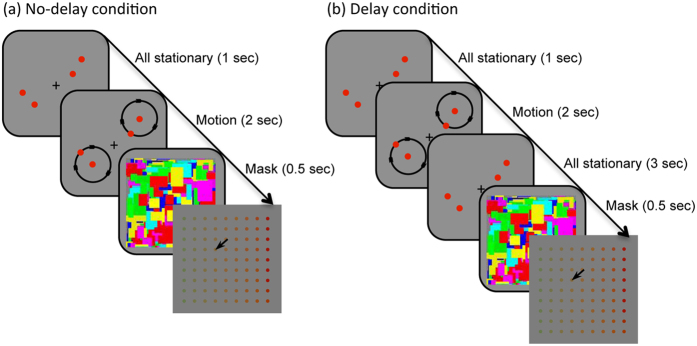
Schematic diagram of the stimulus presentation in the No-delay condition (**a**) and in the Delay condition (**b**).

To test this hypothesis, we compared the changes in color appearance of the following two conditions in Experiment 2. In the no-delay condition, the stimuli were turned off at the same time with the motion offset (Fig. 2a), which was identical to the setting in Experiment 1. In the delay condition, the stimuli remained on the screen for 3 seconds after the motion offset, so that local adaptation to the stationary dot continues after the motion offset (Fig. 2b). If the sensitivity reduction accompanied by local adaptation was the main cause of the color shift, the appearance of the stationary dot would get even more de-saturated.

**Figure 3:**
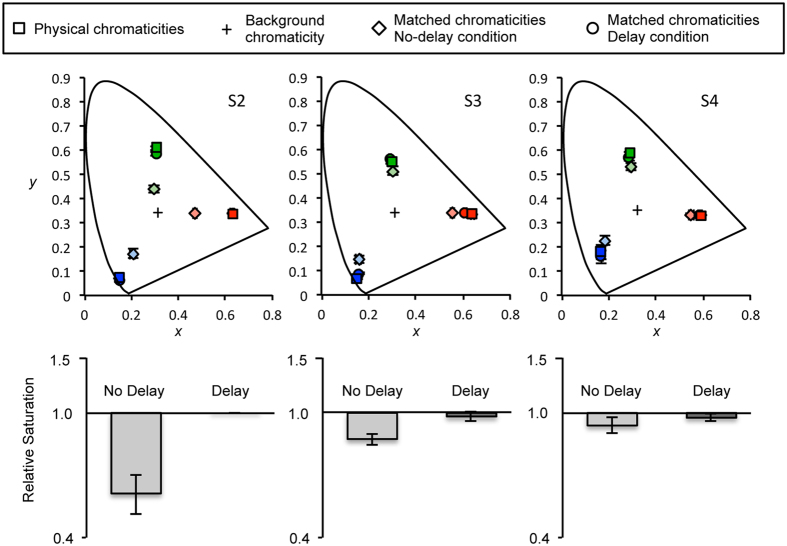
Top: Color matches for stationary dots from three observers shown in CIE color space. Squares represent the matched chromaticities of the dots without motion (baseline). Diamonds represent the matched chromaticities of the stationary dots while physically identical dots are orbiting around the stationary dots. Circles, most of which are overlapped with the squares, represent the matched chromaticities of the dots 3 seconds after motion offset. Error bars, most of which are smaller than symbols, represent ±1 standard error. Bottom: Each bar represents the saturation level of matched colors of the stationary dot relative to the baseline measurements (log-scale). Values below 1 represent a desaturation effect. The measurements of 3 colors were combined for each participant. Error bars represent ±1 standard error for all three colors.

We observed that the appearance of the stationary object shifted back to the original saturated color over time when the object orbiting around the stationary object stopped its motion, even though the amount of local adaptation of that stationary object kept amounting (circles in Fig. 3). To show the de-saturating effect of contextual motion clearly, the relative saturation of the stationary dot compared to the baseline measurements (squares in Fig. 3) was calculated. The measured chromaticity values in CIE color space were transformed to hsv-values (hue, saturation and brightness), then the s-values (saturation) were used for analysis. The bottom plots show that the color of the stationary dot appears less saturated (values lower than 1) when measured immediately after the motion offset (‘No Delay’ condition with light gray bars). However, the saturation level shifted back to the baseline (values close to 1) when measured after a 3-second delay (‘Delay’ condition with dark gray bars). A linear mixed-effect model with a factor of stimulus condition (Delay, No Delay and Baseline) applied to the saturation values, yielding a significant effect of the stimulus condition (*X*^*2*^(2) = 54.354, *p* < 0.0001). A Tukey post hoc test revealed that the effect was driven by the large desaturation of the No Delay condition (Delay – Baseline, *p* = 0.955; No Delay – Baseline, *p* < 0.0001; No Delay – Delay, *p* < 0.0001). If local adaptation and consequent sensitivity reduction were responsible for the de-saturation of the stationary object, the color appearance of the stationary object in the delay condition should have been de-saturated to a greater extent. Nevertheless, we found that color appearance shifted back to its original color appearance in the delay condition, indicating that local adaptation of the stationary object cannot explain the motion-induced color shift.

### Motion vs. transient neural signals at multiple retinal locations

The results of the previous experiments show that kinetic context modulates color appearance of a stationary object. However, does the motion-specific neural response cause the shift in color appearance of the stationary object? Or, alternatively, are the transient neural signals induced by the moving object responsible for the shift in color appearance? The latter can be a plausible alternative hypothesis because transient neural responses are triggered for the neurons at the corresponding retinotopic locations with an object’s displacements due to motion. Consistently, a previous study showed that presentation of a black flickering dot at multiple, random locations around a stationary dot was potent enough to cause a large shift in brightness of the stationary dot, although a flickering dot at one fixed location did not induce brightness shift^22^. However, this pervious study cannot conclusively dissociate the effect of the transient neural signals from the effect of motion-specific neural signals as a main cause of the shift in color appearance, because the flicker of a dot at multiple locations also induces strong motion-specific neural signals due to apparent motion. In Experiment 4, we tested the transient signal hypothesis by directly comparing an apparent-motion condition and a multi-flicker condition where flickering dots at multiple locations did not induce apparent motion perception.

**Figure 4:**
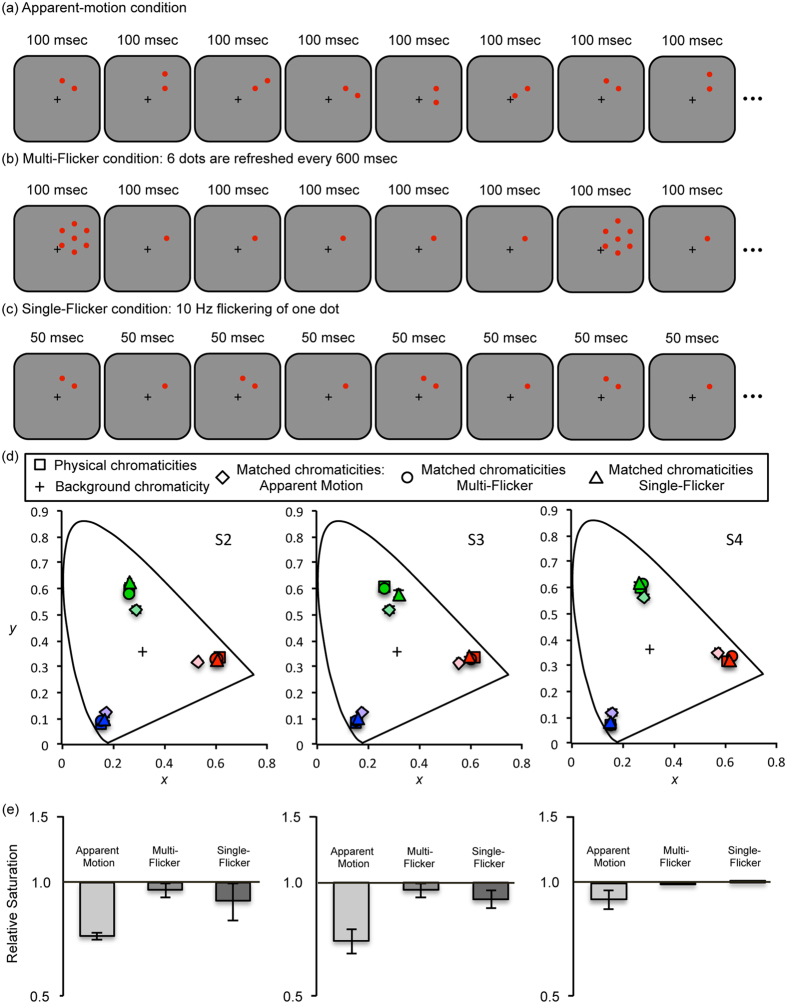
(**a**) Schematic illustration of the stimulus presentation in the apparent-motion condition. A single dot was flashing at six different locations along an invisible circular trajectory, which resulted in perception of apparent motion. (**b**) Schematic illustration of the stimulus presentation in the multi-flicker condition. Six identical dots were flashing every 600 msec at the fixed location. (**c**) Schematic illustration of the stimulus presentation in the single-flicker condition. A single dot was flashing at the same location at 10 Hz. (**d**) Color matches for the stationary dot from three observers shown in CIE color space. Squares represent the matched chromaticities of the dots without flickering or motion (baseline). Diamonds represent the matched chromaticities of the stationary dot in the apparent-motion condition. Circles, most of which are overlapped with the squares, represent the matched chromaticities of the stationary dot in the multi-flicker condition. Triangles, most of which are overlapped with the squares, represent the matched chromaticities of the stationary dot in the single-flicker condition. Error bars, most of which are smaller than symbols, represent ±1 standard error. (**e**) Each bar represents the saturation level of matched colors of the stationary dot relative to the baseline measurements (log-scale). Values lower than 1 represent a desaturation effect and values larger than 1 indicate a higher saturation compared to the baseline condition. The measurements of 3 colors were combined for each participant. Error bars represent ±1 standard error for all three colors.

In the apparent-motion condition, a single dot was presented for 100 msec at one of the 6 different pre-determined locations and the single dot was displaced over the next location every 100 msec resulting in the perception of apparent motion (Fig. 4a). In the multi-flicker condition, a stationary dot was surrounded by 6 dots that were presented for 100 msec at every 600 msec (Fig. 4b). The magnitude of neural transients triggered by the onset and offset of the surrounding dots at 6 locations is equivalent over time between the apparent-motion and multi-flicker conditions. Nevertheless, the motion-specific neural responses are elicited by the apparent motion condition while they are absent in the multi-flicker condition. We also tested the single-flicker condition, in which a single dot was flickering (10 Hz) at one of the 6 pre-determined locations (Fig. 4c). The number of onset and offset in the single-flicker condition was matched to the apparent-motion condition, but the flicker occurred at a fixed location, which resulted in the absence of motion perception.

Color appearance of the stationary dot, measured by the memory-based choice task, revealed the critical role of motion in the original observation. When transient neural signals were triggered by flickering dots at six retinotopic locations (multi-flicker condition), the appearance of the stationary dot (circles in Fig. 4d) did not change from its appearance measured in the absence of any transient signals in the surround (baseline, squares in Fig. 4d). The shift in color appearance was not observed in the single-flicker condition either, in which a single dot was flickering at one of the 6 locations (triangles in Fig. 4d). However, when the perception of apparent motion was accompanied by the sequentially presented single dot along six different locations, a clear shift in color appearance of the stationary dot was observed (diamonds in Fig. 4d). The relative saturation of the stationary dot in the apparent motion condition was lower than that in multi-flicker and single-flicker conditions (Fig. 4e). A linear mixed-effect model applied to the saturation value yielded a significant effect of the four stimulus condition (*X*^*2*^(3) = 118.76, *p* < 0.0001). A Tukey post hoc test revealed that the effect was largely driven by the desaturation of the apparent-motion condition (apparent-motion – baseline, *p* < 0.001; multi-flicker – apparent-motion, *p* < 0.001; single-flicker – apparent-motion, *p* < 0.001; multi-flicker – baseline, *p* = 0.5904; single-flicker – baseline, *p* = 0.0049; single-flicker – multi-flicker, *p* = 0.1667). This result suggests that motion-specific neural responses evoked by the kinetic context, rather than transient neural signals triggered by the stimulus onset and offset, induce the shift in color appearance of the stationary object.

### Attentional modulation of neural response

Previous experiments show that motion-specific neural responses, rather than transient neural signals or local adaptation, mediate the motion-induced color shift. In Experiment 4, we examined whether the motion-induced color shift was mediated by attention. Attention can also explain the motion-induced color shift without assuming interaction between color and motion information. Specifically, attention modulates neural responses of the same stimulus^23^,^24^, even resulting in different perceptual experiences^25^,^26^. In the context of motion-induced color shift, when attention is directed to the more salient moving dot, neural response of the relatively unattended stationary dot can be attenuated and, consequently, its appearance can become less saturated.

We tested this hypothesis by capitalizing on the chromatic selectivity, commonly observed in various contextual processing. Chromatic gain control mechanisms, a type of normalization, are known to be chromatically selective^15^. Collinear facilitation is reduced when different chromaticity is assigned to the flanker stimuli from that of the target^27^. Even visual crowding is nearly abolished when different colors are assigned to the target and surrounding stimuli^28^. Thus, we hypothesized that the motion-induced color shift should be attenuated, if not abolished, when chromatically selective interaction is disrupted by setting different colors for the stationary and moving dots. On the other hand, if attentional modulation of neural responses is responsible for the motion-induced color shift, the color appearance of the stationary dot should change irrespective of the color of the moving dot.

**Figure 5:**
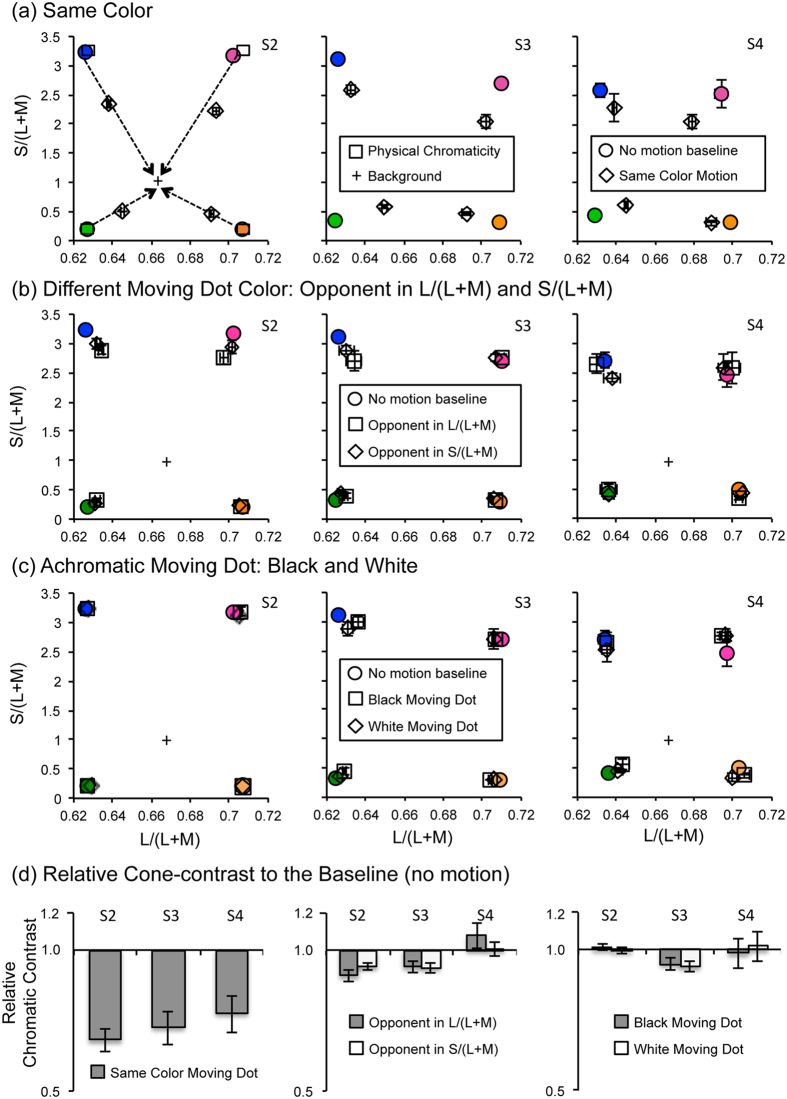
Measured color appearances of the stationary dots is shown in cone-excitation based color coordinates. In the diagram, the x-axis represents L/(L + M), indicating L-cone contrast, and the y-axis represents S/(L + M), indicating S-cone contrast. Data from three individual observers are shown in each panel. (**a**) Perceived color appearance of the stationary dots without (circles) and with (diamonds) moving dots in the surround. Squares represent the physical chromaticities of the dots. Four arrows represent the directions of color appearance shift, which were consistently toward the chromaticity of the gray background. (**b**) Perceived color appearance of the stationary dots when the moving dots’ chromaticity were opponent either in L-cone contrast (squares) or in S-cone contrast (diamonds). Circles represent the matched color appearance without motion. (**c**) Perceived color appearance of the stationary dots when the moving dots were either black (squares) or white (diamonds). Circles represent the matched color appearance without motion. Error bars, most of them are smaller than symbols, represent ±1 standard error. (**d**) Relative cone-contrast of the stationary dot with contextual motion to the no-motion baseline (left: same color, middle: opponent color, right: achromatic). Error bars represent ±1 standard error for all four colors.

To assess the chromatic selectivity of the motion-induced color shift, four chromaticities were chosen in terms of cone-contrast (squares in Fig. 5a). For each color of the stationary dot, we tested whether the moving dot with the same color, with the opponent color in L-cone contrast, and with the opponent color in S-cone contrast could induce a large shift in color appearance of the stationary dot as observed in the previous experiments. In addition, we tested whether the motion-induced appearance shift can occur between chromatic and luminance channels by using a colored stationary dot and an achromatic moving dot. This test with cone-contrast based colors also allowed us to interpret the color appearance shift in terms of the changes in chromatic contrast, rather than the changes in perceived saturation.

When chormaticities of the stationary and moving dots were identical, the color appearance of the stationary dot shifted in the direction of reduced chromatic contrast (diamonds in Fig. 5a) compared to the appearance of the dot without a moving dot around it (baseline condition, circles in Fig. 5a). To confirm the shift in color appearance, the averaged cone contrast values (both L- and S-cone contrast) in each condition relative to the baseline condition (Fig. 5d) were subject to a linear mixed-effect model analysis. The model prediction with a factor of the stimulus condition revealed that the contextual motion of the same colored dot yield a significant difference in relative to the baseline (*X*^*2*^*(1)* = 121.35, *p* < 0.0001). However, the motion-induced color shift was largely attenuated when the chromatic polarity of the moving dot was set differently from the stationary dot (Fig. 5b, *X*^*2*^*(2)* = 4.655, *p* = 0.09754) and was virtually abolished when the moving dot was achromatic (Fig. 5c, *X*^*2*^*(2)* = 1.4483, *p* = 0.4847).

This color-specific influence of motion on color appearance is inconsistent with the attentional account, which does not predict color specificity. Further, the reduction in chromatic contrast of the stationary dot suggests that chromatically selective normalization (i.e., chromatic contrast gain control) induced by contextual motion may mediate the motion-induced color shift.

### Neural normalization as a possible mechanism

How does simple motion of an object in context change the color appearance of another, identical object? The chromatic contrast gain control mechanism is thought to occur by normalizing neural responses of an object and its context. Similarly, chromatically selective contrast reduction observed in the previous experiment suggests that a normalization process could also be involved in the motion-induced appearance shift. If a process normalizes neural responses of the moving and stationary objects where the neural responses of the moving object are greater than the stationary object due to the motion signal, it enhances the gain of the moving object and reduces the gain of the stationary object^29^, which may consequently affect the appearance of the moving dot as well as the stationary dot.

We tested the prediction of normalization by manipulating the physical saturation level of the stimuli. All colors tested in the previous experiment were close to the maximum saturation and, thus, the changes in saturation of the moving dot were difficult to measure. In Experiment 5, therefore, de-saturated red (*x* = 0.528, *y* = 0.340), green (*x* = 0.285, *y* = 0.508) and blue (*x* = 0.192, *y* = 0.137) were used for the stationary and moving dots. This insured that possible appearance shifts in the moving dot also became observable. After the offset of the stimulus presentation, participants were heard either a high-pitch or a low-pitch tone indicating which dot the participants should report on (i.e., high pitch: stationary dot, low pitch: moving dot). The tone was presented after the stimulus presentation to prevent attentional allocation to a specific dot during the stimulus presentation. We also obtained two baseline conditions where appearance of a single stationary dot was measured without a moving dot and the appearance of a single moving dot was measured without a stationary dot.

**Figure 6:**
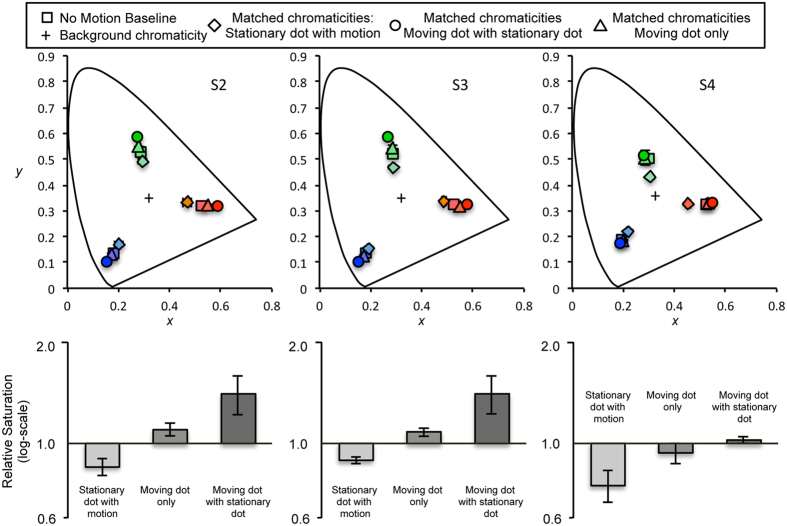
Top: Color matches for the stationary and the moving dot from three observers shown in CIE color space. Squares represent the matched chromaticities of the dots without motion (baseline). Diamonds represent the matched chromaticities of the stationary dot. Circles represent the matched chromaticities of the moving dot. Triangles represent the matched chromaticities of the moving dot when there was no stationary dot (moving baseline). Error bars, most of which are smaller than symbols, represent ±1 standard error. Bottom: Each bar represents the saturation level of matched colors of the stationary dot relative to the baseline measurements (log-scale). Values lower than 1 represent a desaturation effect and values larger than 1 indicate a higher saturation compared to the baseline condition. The measurements of 3 colors are combined for each participant. Error bars represent ±1 standard error for all three colors.

The same moving dot appeared more saturated when accompanied with a stationary dot (circles Fig. 6) compared to when it was presented without the stationary dot (triangles Fig. 6). The appearance of the stationary dot (diamonds Fig. 6) shifted to appear less saturated than the baseline condition in which the appearance of the stationary dot was measured in isolation (baseline, squares Fig. 6). The bottom plots in Fig. 6 show the relative saturation of the stationary and moving dots in comparison to the saturation-level of the stationary dot in the no-motion baseline condition. When both moving and stationary dots were present, a saturating effect in the moving dot (darkest gray bars) and a de-saturating effect in the stationary dot (lightest gray bars) were observed, although the saturating effect was virtually absent for one subject. A linear mixed-effect model applied to the saturation value yielded a significant effect of the four stimulus condition (*X*^*2*^*(3)* = 97.532, *p* < 0.0001). A Tukey post hoc test revealed that all pairs were different (all *ps* < 0.0001) except the two baseline conditions (moving dot only vs stationary dot only, *p* = 0.926), indicating that there was no substantial effect of motion when only a moving dot was present.

The opposite direction of appearance shift in the stationary and moving dot is consistent with the qualitative prediction of normalization. However, a continuously moving dot is free from local adaptation, which may cause the saturating effect in the moving dot. Specifically, the moving dot can appear more saturated because local adaptation of the moving dot is effectively reduced and transient neural responses are continuously elicited along its motion path^30^. However, the appearance shift in moving dot alone condition was inconsistent among observers, and the magnitude of this shift was not comparable to the magnitude of the color appearance shift observed when both the stationary and moving dots were present. This result indicates that interaction between stationary and moving objects is a critical determinant of the motion-induced appearance shift for both stationary and moving objects.

## Discussion

Does a tennis ball appear to be the same saturated yellow when it is sitting on a table alone compared to when there is another, identical ball rolling around it? The experiments reported here indicate that, “it does not”. The motion-induced color shift demonstrates that neural representation of the color of a stationary object is modulated by nearby moving objects with the same color. We establish that the motion-induced color shift reflects the interaction between color and motion information by ruling out alternative accounts based on local adaptation (Experiment 2), transient neural signals (Experiment 3) and attention (Experiment 4). Taken together, these results suggest that kinetic context as well as chromatic context can determine the color appearance of an object.

We previously proposed that normalization (i.e., the contrast gain control mechanism) triggered by object motion can change the brightness of both moving and stationary objects^22^. The normalization operates at multiple stages of visual processing, including the retina and visual cortices^29^. The divisive normalization model predicts that the neural response of a moving object increases and the neural response of a stationary object decreases when both the stationary and moving objects are present in a visual scene. Consequently, the moving dot should appear more saturated while the stationary dot should appear de-saturated. In Experiment 5, we showed that a stationary dot appeared de-saturated while a moving dot appeared more saturated compared to the baseline conditions in which only one stationary dot was presented without a moving dot. This opposite direction of color shift between the stationary and moving dots is consistent with the prediction based on the divisive normalization model^22^.

The chromatically selective induction from contextual motion (Experiment 4) is also consistent with the prediction based on the chromatic contrast gain control mechanism^15^, which is a type of neural normalization. Stimulation of the suppressive surround of cortical neurons is chromatically selective^31^. The responses of neurons in V1 and V2 declined with increasing stimulus size covering their suppressive regions. However, if the surrounds had different chromaticity from the central region, the surrounds did not modulate the response properties of those neurons, indicating the cortical origin of the chromatic selectivity. We also found that the color shift due to contextual motion is still observed when a stationary dot is presented to one eye and a moving dot is presented to the other eye (Supplementary Figure S1), further suggesting that the origin of this neural process is cortical.

One may argue that the motion-induced appearance shift is mediated by different mechanisms because there is no reason to suppose that the moving dot stimulates the suppressive region of the neuron rather than stimulates neurons with neighboring receptive fields. Perceptual grouping could have played an important role by forming a higher-order representation of stationary and moving objects of the same color^32^,^33^. Specifically, those stationary and moving dots of the same color are perceptually grouped and, thus, they are perceptually organized as a single object stimulating the center and surrounding regions, similar to a stimulus at the center and an annulus stimulus at the surround.

The motion-induced color shift also contributes to theories of conscious visual awareness. A similar competitive interaction between stationary and moving objects can result in perceptual disappearance, as compellingly demonstrated in motion-induced blindness (MIB). In contrast to the motion-induced color shift, both attention and local adaptation are critical in inducing the perceptual disappearance observed in MIB^34^,^35^. Although it is still unknown how similar competition sometimes leads to changes in appearance but at other times leads to perceptual disappearance, our findings demonstrate that first, the human visual system does not always operate in a winner-take-all fashion upon competition, and second, the sensory mechanism mediating this tight coupling of an object’s appearance and motion would be crucial in determining conscious experience of an object within dynamically changing environments.

## Methods

### Observers

Three observers (S2, S3 and S4) who were naïve to the purpose of the experiments and had normal or corrected-to-normal vision and normal color vision participated in Experiment 2 to Experiment 5. Two observers (S1 and S2) participated in Experiment 1, and S1 was one of the authors, who were excluded from all other experiments. Typical psychophysical studies that concern low-level visual processing require a small number of participants because between-subject variation is low and the scientific conclusions are mainly drawn based on within-subject comparisons between different experimental conditions^36^. No data was excluded from the analysis. All participants provided the informed consent approved by the Florida Atlantic University Institutional Review Board (IRB). The experiments were conducted according to the principles laid down in the Helsinki Declaration, and the experimental protocols were approved by the Florida Atlantic University IRB, which includes the Ethics Committee.

### Stimuli

Stimuli were presented on a Sony CPD-G520, 21” CRT monitor (100 Hz frame rate) and the collection of behavioral responses was controlled by the Psychophysics Toolbox^37^,^38^. The stimuli were presented in a dark room to observers who were positioned 90 cm from the CRT monitor whose R, G, and B guns were calibrated using a spectroradiometer (Ocean Optics USB4000), a light meter (IL-1700) and a luminance meter (Minolta LS100), creating a color look-up-table (1000 levels for each R, G, and B guns). Pairs of the stationary and moving dots (diameter 0.25°) were presented (Fig. 1, top-left,) against an achromatic (gray) background. Luminance, light intensity, of the dots and background was individually set at each observer’s equiluminant level at 10 cd/m^2^ using a minimum motion technique^39^. Either one or two pairs of dots were presented in all experiments to reduce subjects’ gaze-shifts, except Experiment 4 where one stationary dot and six flickering dots were presented. The stationary dots were presented 2° (in visual angle) away from the fixation and each moving dot was presented 1° away from the paired stationary dot. We have chosen the 2° eccentricity for the stationary dot based on the previous study^22^ showing that the effect of contextual motion reliably occurred when the stationary dot was presented at a location larger than 1° in eccentricity. The positions of stationary dot(s) were randomized in each trial while keeping the eccentricity. The speed of the moving dot(s) was 1 cycle/sec in the counterclockwise direction and initial position(s) of the moving dot(s) was also randomized in each trial. The speed of the moving dot(s) was also determined based on the previous study^22^, which showed that the effect of speed was saturated when the speed reached 1cycle/sec. The 9 by 9 array of color patches for the memory-based choice task was presented in the middle of the screen 500 msec after the offset of the stimulus presentation (Fig. 1, top-right). The distance between each dot was about 1° in visual angle, so that the whole array was about 8 by 8 degrees in visual angle.

In Experiment 1, 2 and 3, three basic colors (Red, Green and Blue) were chosen to show that color appearance shift due to motion in context could be observed with any general color. The chromaticity value for each color was defined based on CIE color diagram^40^ with a fixed luminance of 10 cd/m^2^ (squares in Fig. 1, red: x = 0.627, y = 0.344; green: x = 0.276, y = 0.622; blue: x = 0.152, y = 0.074). The 9 by 9 array of color patches for the memory-based choice task was created by systematically varying chromaticity values (x and y in CIE color coordinate). For example, when we measured the color appearance shift for the ‘red’ target, the chromaticity value of each color patch was defined by combining one x-value from nine x-values between the x-value of ‘red’ (x = 0.627) and x-value of gray (x = 0.312), as well as one y-value from nine y-values between the y-value of ‘red’ (y = 0.344) and y-value of gray (y = 0.328).

In Experiment 4, the target colors were chosen based on the cone-excitation-based color space^41^ to investigate neural substrates of the color shift induced by contextual motion. By using cone-excitation-based color space, we manipulated the chromaticities of the stimuli in terms of post-receptoral chromatic channels. Four colors defined by cone-contrast were tested (squares in Fig. 5): [L/(L + M) = 0.707, S/(L + M) = 3.3], which appeared magenta, [L/(L + M) = 0.707, S/(L + M) = 0.2], which appeared orange, [L/(L + M) = 0.627, S/(L + M) = 3.3], which appeared blue, and [L/(L + M) = 0.627, S/(L + M) = 0.2], which appeared green. The chromaticities of 9 by 9 patches for the choice task was defined in the same way described earlier, using 9 L/(L + M) values between each target color’s L/(L + M) value and the L/(L + M) value of gray [L/(L + M) = 0.667], as well as 9 S/(L + M) values between each target color’s S/(L + M) value and the S/(L + M) value of gray [S/(L + M) = 1.0].

### Procedure

Changes in perceived color appearance were measured by a memory-based choice task. Each trial began with the presentation of one or two pairs of stationary dots. After 500 msec of the stimulus onset, one of the dots in each pair began orbiting around the other stationary dot and both the stationary and moving dots were presented for 2 or 3 seconds. Observers were instructed to fix their eyes to the fixation-cross presented in the center of the display during the stimulus presentation. A Mondrain-like color pattern, composed of 40 randomly colored rectangular patches, was presented for 500 msec after the stimulus offset to prevent a color after image. Then, an array (9 by 9) of color patches was presented. Observers chose one of the patches that appeared closest to the appearance of the stationary dot. The choice array was presented until the observers’ choice was made. In Experiment 2, a pair of stationary and moving dots was presented for two seconds, and then the stimuli remained for 3 seconds after the moving dot stopped its motion (became stationary again). After the 3-second delay period, a color-pattern mask was presented for 500 msec, then the presentation of the color choice was followed. Participants were instructed to choose the most similar color to the appearance of the originally stationary dot at the last moment of the stimulus presentation. In all experiments, the appearance of the stationary dot was measured 5 times for each color pair.

### Statistical Analyses

For all statistical analyses, a linear mixed-effect model was applied with a different intercept for each participant. We constructed two models – one with the factor of interest and the other without the factor and then compare those two models with a likelihood-ratio test^42^.

## Additional Information

**How to cite this article**: Hong, S.-W. and Kang, M.-S. Motion Alters Color Appearance. *Sci. Rep.* **6**, 36272; doi: 10.1038/srep36272 (2016).

**Publisher’s note:** Springer Nature remains neutral with regard to jurisdictional claims in published maps and institutional affiliations.

## Supplementary Material

Supplementary Video S1

Supplementary Information
