# Supplementary material for: Motion Alters Color Appearance
Source: Sci Rep. 2016 Nov 8;6:36272. doi: 10.1038/srep36272 (PMC5099971; doi:10.1038/srep36272)
Supplement: Supplementary Information [file srep36272-s2.pdf]

Motion Alters Color Appearance

Sang-Wook Hong[1,2] & Min-Suk Kang[3,4]

1. Department of Psychology, Florida Atlantic University, FL, USA
2. Center for Complex Systems and Brain Sciences, Florida Atlantic University, FL, USA
3. Center for Neuroscience Imaging Research (CNIR), Institute for Basic Science (IBS), Suwon, Republic of Korea
4. Department of Psychology, Sungkyunkwan University, Seoul, Republic of Korea

Supplementary Materials

Running head: Motion-induced Color Shifts

Correspondence from the editor/publisher should be addressed to:

Sang Wook Hong
Department of Psychology
Florida Atlantic University
777 Glades Rd.
BS 12, Room 209
Boca Raton, 33431
561-297-2905 (telephone)
561-297-2160 (fax)
shong6@fau.edu (email)

Supplementary Video caption

Supplementary Video S1 (file name: Hong&Kang_demo_rgb.avi). At the beginning of the demo, a fixation cross appears in the middle of the display window. To observe the illusion best, please fixate eyes to the cross throughout the demo although slight eye movement does not abolish the illusion. Two pairs of dots (all stationary) are presented for one second, and then one of the dots in each pair starts orbiting around the other stationary dots. Color appearance of the stationary dots (highly saturated red, green and blue in serial order) shifts and appears as de-saturated colors right after the motion onset.

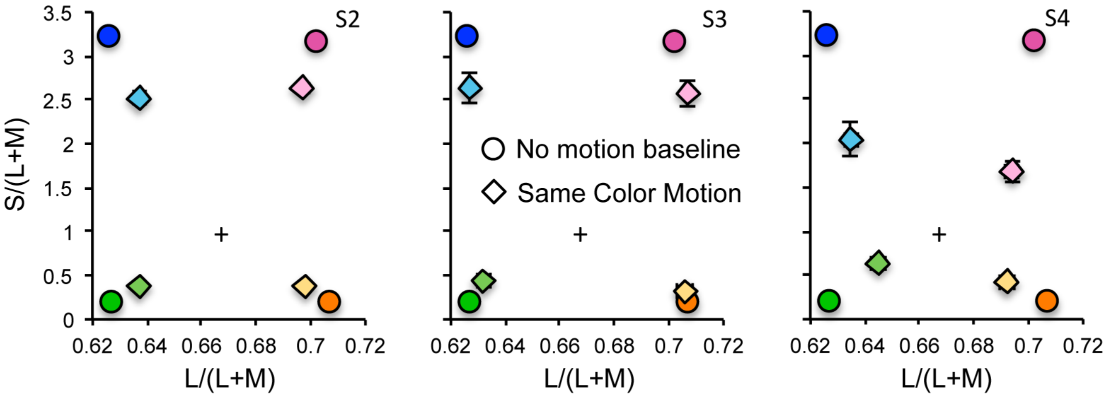

Supplementary Figure S1. Measured color appearance of the stationary dots is shown in cone-excitation based color coordinates. Data from three individual observers are shown in each panel. Circles represent color appearance without motion in the surrounding context. Diamonds represent color appearance of a stationary dot when a moving dot is presented to the other eye. Error bars, most of which are smaller than symbols, represent ±1 standard error.
